# Pharmacological Responses of the G542X-CFTR to CFTR Modulators

**DOI:** 10.3389/fmolb.2022.921680

**Published:** 2022-06-24

**Authors:** Xinxiu Fang, Jiunn-Tyng Yeh, Tzyh-Chang Hwang

**Affiliations:** ^1^ Dalton Cardiovascular Research Center, University of Missouri, Columbia, MO, United States; ^2^ School of Medicine, National Yang Ming Chiao Tung University College of Medicine, Taipei, Taiwan; ^3^ Department of Pharmacology, National Yang Ming Chiao Tung University College of Medicine, Taipei, Taiwan

**Keywords:** CFTR, premature termination codon, nonsense mutations, CFTR correctors, read-through reagent

## Abstract

Cystic fibrosis (CF) is a lethal hereditary disease caused by loss-of-function mutations of the chloride channel cystic fibrosis transmembrane conductance regulator (CFTR). With the development of small-molecule CFTR modulators, including correctors that facilitate protein folding and expression and potentiators that promote channel activity, about 90% of the CF patients are now receiving efficacious target therapies. G542X-CFTR, a premature termination codon (PTC) mutation, is the most common disease-associated mutation found in the remaining 10% of patients that await effective drugs to rectify the fundamental defects caused by PTC. In this study, we employed biophysical and biochemical techniques to characterize the pharmacological responses of the translational products of G542X-CFTR to a range of new CFTR modulators. Specifically, we identified two different proteins translated from the G542X-CFTR cDNA using western blotting: the C-terminus truncated protein that responds to the C1 corrector which binds to the N-terminal part of the protein and a full-length CFTR protein through the read-through process. Electrophysiological data suggest that the read-through protein, but not the C-terminus truncated one, is functional and responds well to CFTR potentiators despite a lower open probability compared to wild-type CFTR. As the expression of the read-through products can be increased synergistically with the read-through reagent G418 and C1 corrector, but not with combinations of different types of correctors, we concluded that an efficacious read-through reagent is a prerequisite for mitigating the deficits of G542X-CFTR. Moreover, the CFTR potentiators may help improve the effectiveness of future combinational therapy for patients carrying PTCs such as G542X.

## Introduction

Cystic fibrosis (CF) is a life-threatening, autosomal recessive hereditary disease caused by loss-of-function mutations of the *cftr* gene which encodes a chloride channel cystic fibrosis transmembrane conductance regulator (CFTR) (Riordan et al., 1989; O’Sullivan and Freedman, 2009; De Boeck and Amaral, 2016). This 1480-amino-acid protein is comprised of five domains: two transmembrane domains (TMD1 and TMD2), two nucleotide-binding domains (NBD1 and NBD2), and a regulatory (R) domain (Hwang et al., 2018). To date, more than 2000 CFTR variants including 383 CF-causing mutations have been reported (“Cystic Fibrosis Mutation Database,” 2021). These pathogenic mutations can be categorized into six classes based on the molecular mechanisms underlying the defects of CFTR proteins (Boyle and De Boeck, 2013; Wang et al., 2014): Class I, the absence of functional protein production due to a premature stop codon (PTC, e.g., G542X) or other mutations; Class II, defected folding and trafficking of the CFTR protein (e.g., ΔF508); Class III, reduced open probability (or gating defect, e.g., G551D); Class IV, decreased CFTR single-channel conductance (e.g., R117H); Class V, reduced synthesis of the CFTR protein (e.g., A455E); Class VI, reduced stability of mature CFTR proteins in the cell membrane (e.g., Q1412X). However, many mutations have been shown to possess multiple defects. We take the Class VI mutation Q1412X for example. Aside from a decreased stability (Class VI), the Q1412X-CFTR presents a clear gating defect (Class III) (Yeh et al., 2019). A detailed and precise classification is crucial for predicting the clinical outcome and devising corresponding treatment.

Decades of mechanistic and structural studies of the CFTR protein and CF pathogenesis have culminated in the successful development of drugs for CF treatment in the past 10 years. Two types of CFTR modulators have been approved by the FDA for clinical use: CFTR correctors (VX-809, VX-445, and VX-661) which facilitate protein folding and increase the protein expression on the membrane, and CFTR potentiators (e.g., VX-770 and GLPG 1837) which increase the open probability of the protein (Lopes-Pacheco, 2019). The combinations of a potentiator plus a corrector (e.g., Orkambi and Symdeko), or a potentiator plus two types of correctors (e.g., Trikafta) have resulted in the functional synergism of CFTR leading to significant clinical improvement for over 90% of the CF patients (Wainwright et al., 2015; Taylor-Cousar et al., 2017; Middleton et al., 2019; Barry et al., 2021). However, there has not been an approved therapy for the rest 10% of patients including those carrying the second most common mutation G542X-CFTR.

G542X is a Class I mutation caused by the introduction of a premature termination codon (PTC) at position 542, resulting in a decreased mRNA production partly due to the nonsense-mediated mRNA decay (NMD) (McHugh et al., 2018), and the production of two different proteins—a C-terminal truncated CFTR protein and a full-length protein with a missense mutation at position G542 *via* the read-through process (Roy et al., 2016). Our previous study showed that the chloride current carried by the G542X-CFTR is mainly contributed by the read-through protein, and hence proposed that an effective treatment for PTCs should include minimizing the NMD effect, maximizing the read-through process, and improving CFTR expression and function (Yeh and Hwang, 2020). To realize the first two strategies, several reagents have been developed and are currently in the pre-clinic stage (Pinto et al., 2021). In the present study, we focused on investigating the effect of different types of CFTR correctors on the protein expression of G542X-CFTR.

Three types of CFTR correctors have been identified based on their mechanism of action (Veit et al., 2020): type I correctors (C1) such as VX809, VX661, and C1-605 in this work are thought to stabilize the NBD1 and TMD1/2 interface (Molinski et al., 2018; Singh et al., 2020); type II correctors (C2) (e.g., Corr-4a) act on NBD2 and its interfaces (Okiyoneda et al., 2013); and type III correctors (C3) (e.g., VX445) facilitate NBD1 folding (Veit et al., 2020). The expected synergistic action among different CFTR correctors is best demonstrated by the marked clinical effects of a recently-approved drug Trikafta, the first triple combination therapy consisting of a CFTR potentiator and two different types of CFTR correctors. A similar strategy has been investigated for Class I mutations in the lab. For example, a combination of a CFTR potentiator and two types of CFTR correctors showed improvement in the function of W1282X-CFTR, but such a combination exerted little effect on G542X-CFTR (Mutyam et al., 2021). These observations thus underscore the heterogeneity of the PTC mutations in response to CFTR modulators, as the position of the PTC dictates the functional and pharmacological characteristics of the protein (Yeh and Hwang, 2020).

In this study, we investigated the effects of CFTR correctors on the protein expression of G542X-CFTR. Using the antibodies recognizing different motifs in CFTR, we were able to identify and characterize both the C-terminal truncated and full-length read-through G542X-CFTR proteins. For the C-terminal truncated G542X-CFTR, only the Type 1 corrector could increase the protein expression, which is consistent with the structural study showing that Type 1 correctors bind to the TMD1 (Fiedorczuk and Chen, 2022). While the expression of mature G542X-CFTR proteins could be improved with the combination of a read-through reagent and different types of correctors, no clear synergism between the correctors was detected. These results provided insights into the effects of different CFTR correctors on the most common PTC mutation and may serve as a guide for future drug development.

## Materials and Methods

### Materials

For western blot, all equipment and materials including electrophoresis chambers, tank transfer systems, 4%–20% gradient gels, and polyvinylidene difluoride (PVDF) membrane were purchased from Bio-Rad Laboratories (Hercules, CA, United States). Type 1 corrector X281605 and type 2 corrector X289990 were provided by AbbVie Inc. (North Chicago, IL, United States), which are abbreviated as C1-605, and C2-990, respectively, in this work. VX-445 contains both R-VX-445 (N-(1,3-dime-thylpyrazol-4-yl)sulfonyl-6-(3-(3,3,3-trifluoro-2,2-dimethyl-propoxy)pyrazol-1-yl)-2-((4R)-2,2,4-trimethy-lpyrrolidin-1-yl)pyridine-3-carboxamide) and S-VX-445 (N-(1,3-dimethylpyrazol-4-yl)sulfonyl-6-(3-(3,3, 3-trifluoro-2,2-dimethyl-propoxy)pyrazol-1-yl)-2-((4S)-2,2,4 trimethylpyrrolidin-1-yl)pyridine-3-carboxamide) was purchased from Selleck Chemicals (Houston, TX, United States). All the correctors were stored as a 10 mM stock solution in DMSO at −20°C. Read-through agent G418 was purchased from Gibco (Waltham, MA, United States) and stored as a 100 mM stock in DMSO at −20°C. A mouse monoclonal anti-CFTR antibody MM13-4 was purchased from Millipore Sigma (Burlington, MA, United States); the mouse monoclonal anti-CFTR antibody Ab 596 was provided by the Cystic Fibrosis Foundation (Bethesda, MD, United States); the β-actin antibody AC-15 was purchased from Santa Cruz Biotechnology and anti-mouse IgG; and the HRP-linked antibody was bought from Cell Signaling Technology (Danvers, MA, United States).

For immunoprecipitation (IP) experiments, magnetic beads (from Dynabeads™ Protein G Immunoprecipitation Kit with cat. No. 10007D), IP lysis buffer, and ethanolamine (from Pierce™ Direct Magnetic IP/Co-IP Kit with cat. No. 88828), and imidoester crosslinkers DMP (cat. No. 21667) were purchased from Thermo Scientific (Rockford, IL, United States). A ready-to use colloidal Coomassie brilliant blue was purchased from Alfa Aesar (Tewksbury, MA, United States), and sodium borate decahydrate from Millipore Sigma (BuMA, United States). All the chemicals were freshly prepared before use.

For electrophysiology studies, the bath solution was both used in whole-cell and single-channel recordings with the same formulation containing (in mM): 145 NaCl, 5 KCl, 2 MgCl_2_, 1 CaCl_2_, 5 glucose, 5 HEPES, and 20 sucrose, with pH adjusted to 7.4 using 1 M NaOH. The whole cell pipette solution comprises (in mM): 10 EGTA, 10 HEPES, 20 TEACl, 10 MgATP, 2 MgCl_2_, 85 aspartates, 16 pyruvates, and 5.8 glucose, with pH adjusted to 7.4 using CsOH. The pipette solution for inside-out patch-clamp experiments (i.e., standard inside-out perfusate) contains (in mM): 150 mM NMDG-Cl, 2 mM MgCl_2_, 1 mM CaCl_2_, 10 mM EGTA, and 8 mM Tris, pH 7.4 adjusted with NMDG. The inside-out solution used for single-channel recording contains (in mM): 150 NMDG-Cl, 10 EGTA, 10 HEPES, 8 Tris, and 2 MgCl_2_, with pH adjusted to 7.4 using NMDG. PKA and MgATP were purchased from Sigma-Aldrich. MgATP was stored as a 500 mM stocked at −20°C. Forskolin and genistein were purchased from Alexis (Plymouth Meeting, PA, United States) and stored at −20°C. CFTRinh-172, provided by Dr. Robert Bridges (Rosalind Franklin University, Chicago, IL, United States), who directs the chemical compound distribution program sponsored by Cystic Fibrosis Foundation Therapeutics (Bethesda, MD, United States), was stored at −70°C as a 5 mM stock solution in DMSO. GLPG1837 was purchased from Selleck Chemicals (Houston, TX, United States) and stored as a 10 mM stock in DMSO at −70°C. All the chemicals were freshly prepared. For whole-cell recording, the working solutions were diluted with the bath perfusate and the pH was adjusted to 7.4 with 1 M NaOH. For single-channel recording, the reagents were freshly thawed and diluted with the standard inside-out perfusate and the pH was adjusted to 7.4 with NMDG.

### Mutagenesis and Cell Culture

All CFTR mutations were introduced using the Quick-change XL kit (Agilent). In short, the primers for mutations including G542X (c.1624G > T) were designed in-house. The mutant DNA strands were synthesized using PCR with PfuUltra as DNA polymerase according to the manufacturer’s protocol. The template DNA was then digested by Dpn I. The final products were sequenced by the DNA Core Facility at the University of Missouri to confirm the mutation. The Chinese hamster ovary (CHO) cells were cultured in Dulbecco’s modified Eagle’s medium (Gibco, Waltham, MA, United States) containing 10% (v/v) fetal bovine serum (Sigma-Aldrich, St Louis, MO, United States) at 37°C in 5% CO_2_. All the CFTR constructs were transfected and expressed in CHO cells as described in the previous paper (Yeh and Hwang, 2020). Briefly, for the western blot experiments, the CFTR constructs were transfected with X-tremeGENE HP DNA Transfection Reagent (Roche, Basel, Switzerland) into CHO cells and kept at 37°C for 6 h. After transfection, the correctors or read-through agents were added to the medium, and the cells were incubated at 37°C for 40–45 h before harvesting. DMSO (0.1% v/v) was used as volume control. For patch-clamp recordings, CFTR constructs and green fluorescence protein-encoding pEGFP-C3 (Takara Bio) were co-transfected with PolyFect transfection reagent (Qiagen, Valencia, CA, United States) into CHO cells. The cells were incubated at 27°C for 2–6 days after transfection for electrophysiological experiments.

### Western Blotting

The CHO cells were harvested by scraping in ice-cold PBS and then lysed with RIPA buffer (Santa Cruz Biotechnology, Dallas, TX, United States) containing complete protease inhibitor (EDTA-free; Roche, Basel, Switzerland). All the protein extracts were separated on 4%–20% gradient gels (Bio-Rad Laboratories, Hercules, CA, United States) and transferred to PVDF membranes. The membranes were incubated and probed with primary antibody at 4°C overnight according to our prior study (Yeh and Hwang, 2020), including mouse monoclonal anti-CFTR antibody MM13-4 (dilution 1:500), mouse monoclonal anti-CFTR antibody Ab 596 (dilution 1:3000), and β-actin antibody AC-15 (dilution 1:1600) working as a loading control. The membranes were washed and incubated with a secondary anti-mouse IgG, HRP-linked antibody (dilution 1:5000) at room temperature for 1 h. Super Signal West Pico PLUS chemiluminescence substrate (Thermo Fisher Scientific, Waltham, MA, United States) was applied according to the manufacturer’s instructions and chemiluminescent detection was performed by Molecular Imager Chemidoc (Bio-Rad Laboratories), and CFTR protein band density was quantified using the ImageLab software (Bio-Rad laboratories) and normalized with the band density of the β-Actin within the same lane.

Western blot experiments were carried out as in our prior study in Yeh and Hwang (2020) with two exceptions. 1) Western transfer method and equipment were changed in this work. In our previous publication, semi-dry transfer equipment trans-blot turbo transfer system (Bio-Rad Laboratories, California, United States) was adopted. In contrast, a wet transfer tank mini trans-blot electrophoretic transfer cell (Bio-Rad Laboratories) was used for the current study for high efficiency of transferring the low-expression protein to the PVDF membrane (Mahmood and Yang, 2012). 2) Due to that the molecular weight of the truncated protein of G542X-CFTR (∼50 kDa) is much closed to that of β-actin (43 kD), probing these two kinds of protein via corresponding antibodies (Antibody MM13-4 for truncated G542X-CFTR and antibody AC-15 in the same gel may result in an overlap of the signal. Hence, for the experiments using antibody MM13-4 (Figures 3A, 4A), we loaded the two gels with the same volume of samples and ran them in the same tank. One gel was probed with antibody MM13-4 and the other with antibody AC-15.

### Immunoprecipitation

Immunoprecipitation was performed according to published procedures (Jiang et al., 2010; Pankow et al., 2016) and the manufacturer’s instructions using a Dynabeads™ Protein G magnetic beads and crosslinker dimethyl pimelimidate·2 HCl (DMP). Mouse monoclonal anti-CFTR antibody MM13-4 was bound to protein G magnetic beads at 4°C overnight and covalently crosslinked to the beads with 20 mM DMP in sodium borate (0.1 M) pH 9.0 for 30 min at RT. 200 mM ethanolamine pH 8.0 was used to terminate the reaction and antibody bound-beads were washed by BPS and prepared for the next immunoprecipitation reaction. The CHO cells were harvested by scraping, washed with cold PBS, and lysed on ice in lysis buffer [2 × 50 ml, pH 7.4, 0.025 M Tris, 0.15 M NaCl, 0.001 M EDTA, 1% NP40, 5% glycerol, 1 mM EDTA, and cOmplete protease inhibitor (Roche)]. The insoluble material was removed by centrifugation at 4°C for 20 min. The supernatant was transferred to a tube containing the above antibody-bound beads. The mixture solution was incubated overnight with rocking at 4°C. The magnetic beads were washed with IP lysis buffer. The proteins were eluted by glycine buffer (pH = 2.5) or 2X Dilute SDS Sample Buffer (Bio-Rad Laboratories). The immunoprecipitated sample was divided into two halves and extracted by 4%–20% gradient gels. One-half of the gel was probed by western blot using mouse monoclonal anti-CFTR antibody MM13-4 and the other one was stained with Ready-to-Use Colloidal Coomassie brilliant blue to identify the corresponding bands by western blot.

### Mass Spectrometry and Data Analysis

All in-gel protein digestion for mass spectrometry tests and data analysis were elaborated on in our prior publication (Yeh and Hwang, 2020). The gel band sample was minced into 1 mm^3^ pieces and destained by 50 mM ammonium bicarbonate/50% acetonitrile. The protein was reduced by DTT and alkylated by IAA. Then trypsin was added for digestion overnight. The digested peptide was extracted, lyophilized, and resuspended in 10 μl 5/0.1% acetonitrile/formic acid.

One microliter of the suspended peptide was loaded onto a C18 column (20 cm × 75 μm 1.7 μm) with a step gradient of acetonitrile at 300 nl/min. The Bruker nanoElute system is connected to a timsTOF pro-mass spectrometer. LC gradient conditions: the initial conditions were 2%B (A: 0.1% formic acid in the water, B: 99.9% acetonitrile and 0.1% formic acid), followed by a 4.5 min ramp to 17% B. 17%–25% B over 10 min, 25%–37% B over 5.5 min, the gradient of 37% B to 80% B over 2 min, hold at 80% B for 6 min, back to 3% B in 0.5 min, and hold on 3% B for 1.5 min. The total run time was 30 min.

MS data were collected over an m/z range of 100–1700. During MS/MS data collection, each TIMS cycle included 1MS + an average of 10 PASEF MS/MS scans. The raw data were searched using PEAKS (version X+) with the Uniprot Human protein database. The data were searched with trypsin as an enzyme, two missed cleavages allowed; carbamidomethyl cysteine as a fixed modification; deamidation of asparagine and glutamine, oxidized methionine, and phosphorylation (STY) as variable modification. 20 ppm mass tolerance on precursor ions and 0.1 Da on fragment ions.

### Electrophysiology

For whole-cell recordings, the glass pipettes were made from borosilicate glass capillaries (Kimble & Chase, Vineland, NJ, United States) using a two-step vertical micropipette puller (PP-81L; Narishige, Tokyo, Japan) and made resistance of 1–2 MΩ in the bath solution. The pipette was located close to the outlet of a three-barrel perfusion system controlled by a fast solution exchange device (SF-77B; Warner Instruments, Hamden, CT, United States) with a dead time of ∼30 ms (Tsai et al., 2009).

For single-channel studies, the glass pipettes were made from flaming/brown micropipette puller (Model P-97) (Sutter Instrument, United States) and polished to a resistance of 2–6 MΩ in the bath solution with a home-made microforge. The perfusion solution was changed to the inside-out solution after excision when the seal resistance reached >4 GΩ. All the single-channel traces shown in this work were acquired after >18 min of phosphorylation by perfusing with a solution containing 25 IU PKA and 2 mM ATP to allow CFTR to reach a fully phosphorylated state. To enhance the success rate of getting single-channel data in inside-out patches, forskolin (10 μM) was added to the bath solution prior to patching.

### Statistical Analysis

Each experiment was performed at least three times and the data are shown as the mean ± SEM. Statistical analyses accompanied by graphs were prepared by Igor Pro (Wave Metrics, Lake Oswego, OR, United States). Paired and unpaired Student’s *t*-tests assuming equal variance were used for comparisons between the two groups. For western blot, one-way ANOVA followed by the Dunnett was used for multiple comparisons to compare every mean with a control mean. The results were considered statistically significant with *p*-value < 0.05 (*p < 0.05, **p < 0.01).

## Results

### Both C-Terminus Truncated Protein and Read-Through Protein Were Identified in G542X-CFTR With Western Blot

Two different proteins are expected to be produced out of G542X-CFTR: a dominant C-terminus truncated one with a molecular weight of ∼50 kDa and the read-through, full-length CFTR that may or may not be detected in western blot analysis. Nonetheless, as these two proteins share the same N-terminus, they could be recognized in the same gel with a CFTR antibody targeting the N-terminus.

**FIGURE 1:**
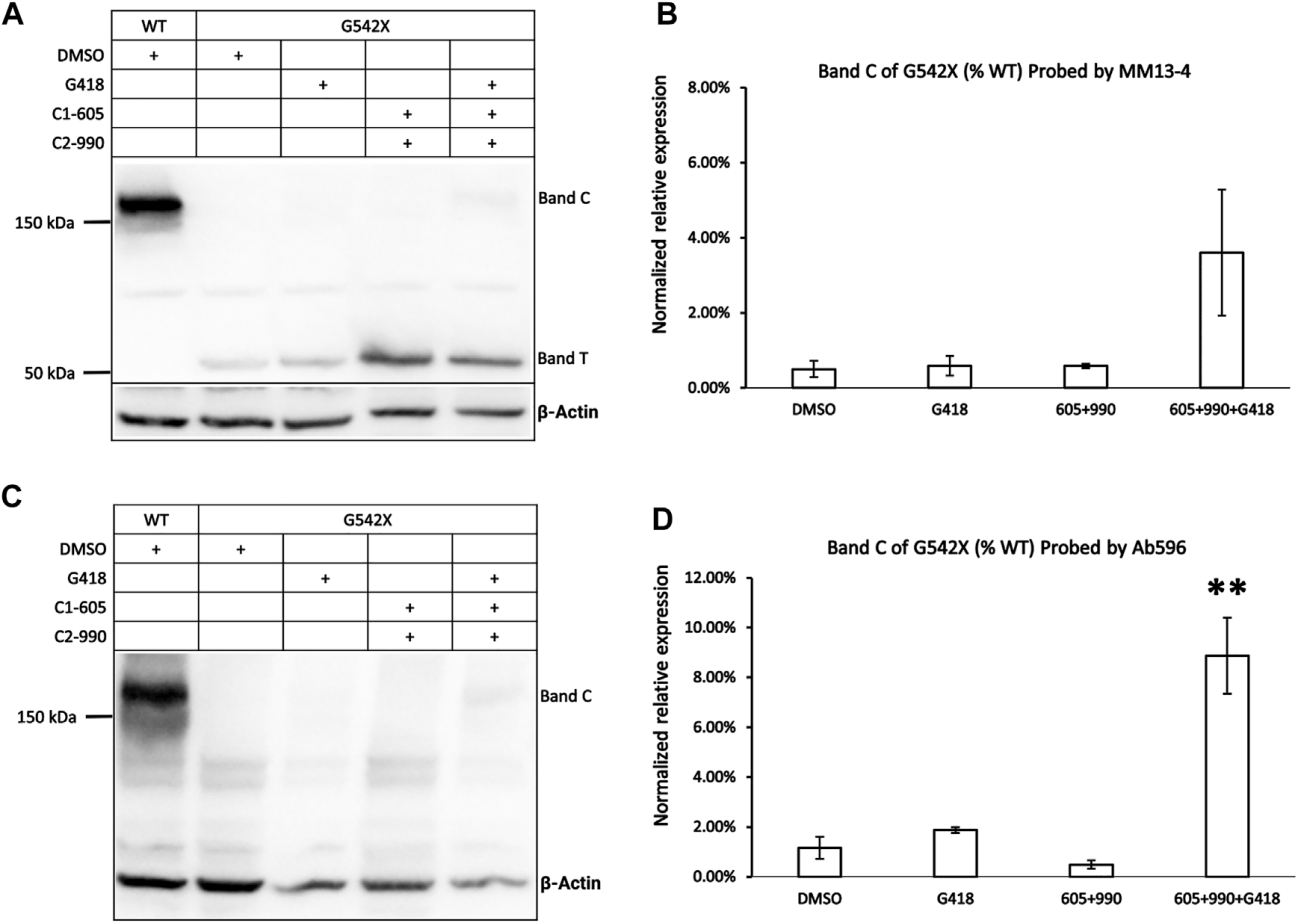
Western blot analysis of G542X-CFTR using two different antibodies. **(A)** Effects of CFTR type 1 corrector 605, type 2 corrector 990, and read-through reagent G418 on G542X-CFTR expression. The gel was probed with CFTR N-terminal antibody MM13-4. **(B)** Quantification of the expression of band C of G542X-CFTR compared to WT-CFTR with MM13-4 (N = 3). **(C)** Western blot of G542X-CFTR similar to panel A except that the gel was probed with antibody 596 which recognizes CFTR’s NBD2. **(D)** Quantification of the expression of band C in G542X-CFTR compared to WT-CFTR by Ab 596 (N = 4). All data are represented as mean ± SEM. The asterisks indicate that the level of band C in G542X-CFTR is significantly higher than that of control in DMSO (ANOVA followed by Dunnett’s test). **p < 0.01.

Figure 1 shows the western blot results of G542X-CFTR treated with the read-through reagent G418, and various combinations of Type 1 corrector (C1-605) and Type 2 correctors (C2-990). When probed with the antibody MM13-4 against the N-terminal a.a. 25–36 of CFTR (Figure 1A), a band with a molecular weight of around 52 kDa could be consistently detected in every sample of G542X-CFTR. This band, labeled band T, is likely the C-terminus truncated G542X-CFTR for several reasons. First, 52 kDa is around the predicted molecular weight of a polypeptide with 541 amino acids. Second, this band was not seen with wild-type (WT) CFTR (Figure 1A). Third, the CFTR antibody 596 recognizing NBD2 successfully detected WT-CFTR but failed to detect this band in the same gel (Figure 1C).

An additional band, labeled band C, with a molecular weight of around 170 kDa was detected by both the antibody MM13-4 (Figure 1A) and Ab596 (Figure 1C) for G542X-CFTR treated with G418 and CFTR correctors. As the molecular weight of band C is compatible with the mature WT-CFTR, this band C likely represents the full-length CFTR protein resulting from the read-through process. The quantitative analysis of the band C in G542X-CFTR showed that the percentages of the read-through protein in G542X-CFTR relative to WT-CFTR band C reached 4%–8% when treated with the combinations of C1 + C2 + G418 (Figures 1B,D). While these combinations of G418 and CFTR correctors significantly increased the level of band C of G542X-CFTR when compared to the control (DMSO), neither G418 alone nor various combinations of C1 and C2 did the same trick (Figures 1B,D). Since Ab596 offered a better signal-to-noise ratio and sensitivity in detecting the band C of G542X-CFTR, we used Ab596 to study the read-through protein (band C) and MM13-4 for the C-terminus truncated protein (band T) in the following experiments.

**FIGURE 2:**
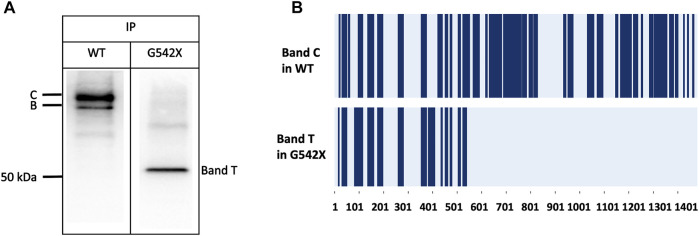
Immunoprecipitation (IP) and mass spectrometry (MS) experiments for the band C of WT-CFTR and the band T of G542X-CFTR. **(A)** Western blots of WT-CFTR and G542X-CFTR after immunoprecipitation with mouse monoclonal anti-CFTR N-terminal antibody MM13-4. Two bands for WT (band C and band B with a molecular weight of ∼170 and ∼150 kDa, respectively), and one band for G542 (band T with a molecular weight of ∼52 kDa) were identified. **(B)** Schematic representation of MS results. The dark blue regions represent the identified peptides. The horizontal axis marks the numbers of the amino acid residues in CFTR.

**FIGURE 3:**
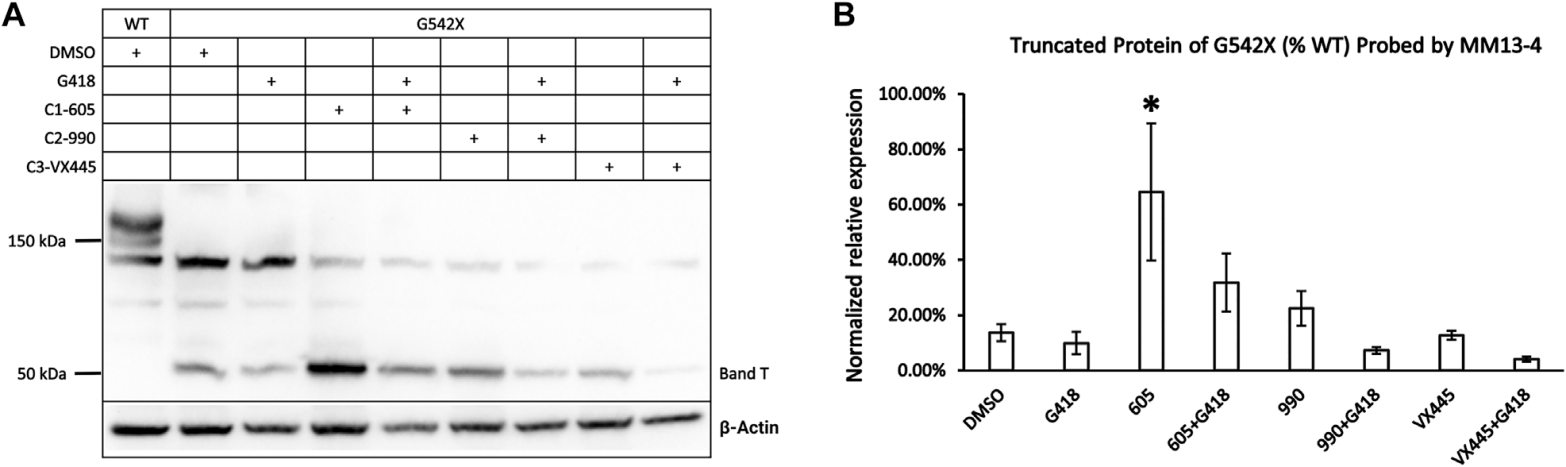
Effects of CFTR correctors on the expression of the C-terminus truncated protein of G542X-CFTR using mouse monoclonal anti-CFTR N-terminal antibody MM13-4. **(A)** Effects of type 1 (605), 2 (990), and 3 (VX-445) CFTR correctors with or without the read-through reagent G418 on G542X-CFTR expression. The gel was probed with anti-CFTR N-terminal antibody MM13-4. **(B)** Quantification of the effects of CFTR correctors on the expression of the C-terminus truncated protein of G542X-CFTR. The band C of WT-CFTR in the same gel was used for normalization. Data are represented as mean ± SEM. The asterisks indicated that the expression of band T in G542X-CFTR is significantly higher when compared with DMSO (N = 3, ANOVA followed by Dunnett’s test). *p < 0.05.

To validate that band T is the C-terminus truncated G542X-CFTR, we used immunoprecipitation to enrich G542X-CFTR band T using antibody MM13-4 (Figure 2A) and cut this band from the gel for mass spectrometric analysis. As shown in Figure 2B, for WT-CFTR band C, the peptides corresponding to segments from the N-terminus to the C-terminus of CFTR were identified. In contrast, no CFTR peptide after L541 could be identified in G542X-CFTR band T, supporting the idea that band T is indeed the C-terminus truncated protein resulting from the premature cessation of the translation due to the introduction of PTC (Keeling and Bedwell, 2011). Of note, a band B with a molecular weight of ∼150 kDa representing the core-glycosylated CFTR was detected in Figure 2A and some other western blotting experiments (e.g., Figures 3A, 6). Since the band B CFTR is immature and non-functional, in this study we focused on characterizing and quantifying the mature and functional band C (Cheng et al., 1990).

### Only Type 1 Corrector Increases the Expression of C-Terminus Truncated Protein of G542X-FCTR

As shown in Figure 1A, the expression of G542X-CFTR band T appeared higher when preincubated with correctors. To differentiate which of the correctors is efficacious for the truncated G542X-CFTR, we examined the effects of C1 corrector 605, C2 corrector 990, and C3 corrector VX-445 (Veit et al., 2020) with or without G418 on the level of band T (Figure 3A) and found that only C1-605 increased the expression of G542X-CFTR band T compared to the DMSO group (Figure 3B). We also noted that co-treatment with G418 decreased the expression of band T of G542X-CFTR (Figures 3A,B) result that may be due to selective toxicity of G418 (Hermann, 2007) or shuffling the translation products more towards the read-through CFTR.

### Possible Binding Site of the Type 1 Corrector 605

A recent cryoelectron microscopy (cryo-EM) study (Fiedorczuk and Chen, 2022) suggested that type 1 correctors VX-809 and VX-661 bind to the TMD1 with the shared structure of 1,3-benzodioxol-5-yl-cyclopropane carboxamide (BCC) headgroup residing in a hydrophobic pocket formed by TM1, 2, 3, and 6. Mechanistically, type 1 correctors may stabilize the partially folded TMD1 in the early biogenesis of CFTR. The observation that only C1-605 significantly increases the expression of C-terminus truncated G542X-CFTR, we hypothesized that C1-605 may share a similar binding site with another type 1 corrector such as VX-809 and VX-661.

**FIGURE 4:**
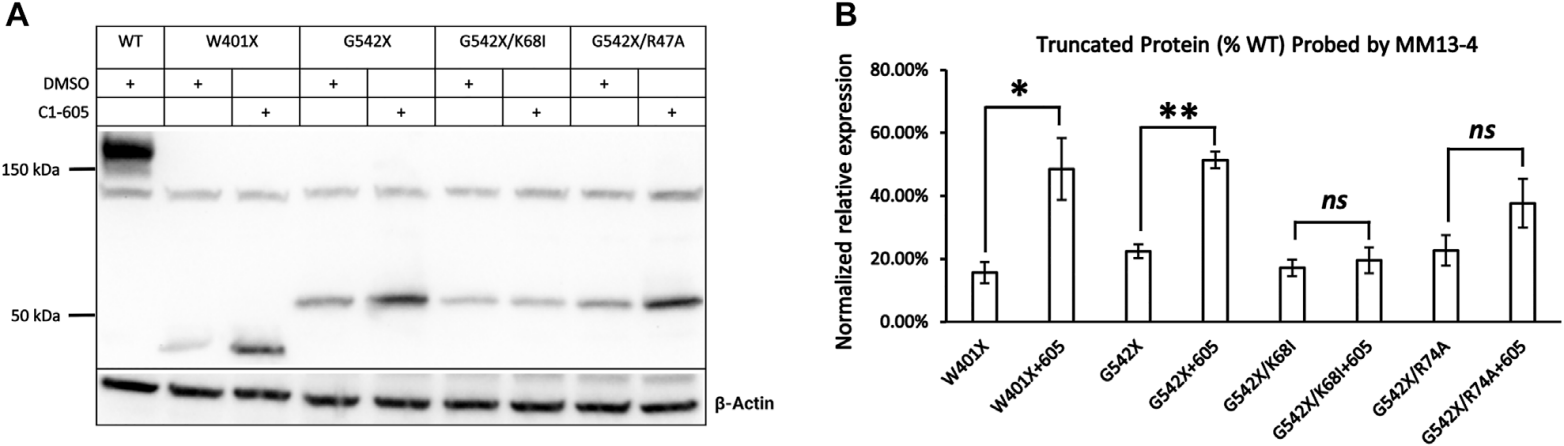
Western blot analysis of the expression of the C-terminus truncated protein of W401X-, G542X-, G542X/K68I-, and G542X/R74A-CFTR. **(A)** Effects of type 1 corrector 605 on the expression of C-terminus truncated protein of W401X-, G542X-, G542X/K68I-, and G542X/R74A-CFTR. The gel was probed with anti CFTR N-terminal antibody MM13-4. DMSO was used as vehicle control for lanes 1, 2, 4, 6, and 9. **(B)** Quantification of the expression of the C-terminus truncated protein of W401X-, G542X-, G542X/K68I-, and G542X/R74A-CFTR compared to WT-CFTR. Data are represented as mean ± SEM. The asterisks indicated a significant increase of the expression in C-terminus truncated protein by type 1 corrector C605 when compared with that of DMSO (N = 3, paired *t*-test). *p < 0.05; **p < 0.01. ns presents no significant increase.

We first tested this hypothesis by examining the effect of C1-605 on W401X-CFTR as this disease-associated PTC mutation is expected to express a C-terminus truncated protein that encompasses the TMD1 (a.a. 1–390) but excludes the majority of NBD1 (a.a. 391–643). If our hypothesis is correct, C1 corrector 605 should increase the expression of the C-terminus truncated protein of W401X-CFTR. Western blotting in Figure 4A showed that for W401X, a band with a molecular weight of about 40 kDa was detected by the antibody MM13-4. The expression of this band whose molecular weight is close to the predicted CFTR truncated with residue 400 was not seen with WT or G542X. Moreover, the expression of this truncated CFTR was increased to about 3-fold by C1-605 (Figure 4B), a magnitude similar to what was seen with G542X-CFTR (Figures 3, 4). These results thus supported the idea that C1-605 binds to TMD1 just like the other two C1 correctors VX-809 and VX-661.

In spite of binding to the same hydrophobic pocket in TMD1, VX-809, and VX-661 interact with different residues due to their distinct chemical structures (Fiedorczuk and Chen, 2022). Specifically, the carboxyl group in VX-809 forms a salt bridge with the side chain of lysine 68 (K68) in CFTR, while an H-bond is formed between the hydroxyl group in VX-661 and the guanidinium group in arginine 74 (R74) in CFTR. To investigate if C1-605 interacts with these residues, we introduced the K68I and R74A mutations in the G542X background and examined whether these mutations affect the effectiveness of C1-605 (Figure 4A). The correcting effect of C1-605 on band T of G542X-CFTR was significantly diminished by the K68I mutation (Figure 4B), suggesting that indeed C1-605 binds to the same pocket with other C1 correctors, and it may possess a similar functional group as VX-809 to interact with residue K68. However, our data with the R74A mutation is not as clear. Although an increase in the expression of band T of G542X/R74A by C1-605 was noted (Figure 4A), this increase failed to reach a statistically significant level probably due to the variation of the data (Figure 4B). A similar increase was seen with the R74A/ΔF508 treated with VX809 compared to DMSO (Fiedorczuk and Chen, 2022), so we cannot decide whether C1-605 forms an H-bond with R74 in CFTR.

### Synergism Between the Read-Through Reagent G418 and CFTR Correctors for the Expression of Mature G542X-CFTR Proteins

**FIGURE 5:**
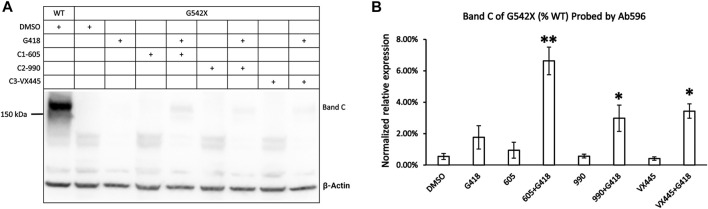
Western blot analysis of the expression of the read-through protein of G542X-CFTR. **(A)** Effects of type 1 (605), 2 (990), and 3 (VX-445) of CFTR correctors with or without the read-through reagent G418 on G542X-CFTR expression. The gel was probed with antibody 596. **(B)** Quantification of the expression of the read-through protein of G542X-CFTR compared to WT-CFTR. The data are represented as mean ± SEM. The asterisks indicated that the expression of the read-through G542X-CFTR protein is significantly increased by combinations of correctors and the read-through reagent G418 when compared with DMSO (N = 3, ANOVA followed by Dunnett’s test). *p < 0.05; **p < 0.01.

The abovementioned experiments show that type 1 corrector 605, by binding to a hydrophobic pocket in the TMD1, can increase the expression of the C-terminus truncated G542X-CFTR. However, the C-terminus truncated G542X-CFTR is not likely to have any function (Yeh and Hwang, 2020). We have deduced that the whole-cell current observed in G542X-CFTR was carried by the read-through product. In the current study, we optimized the western blot protocols and tested several newly developed CFTR correctors in an attempt to quantify the effects of a combination regimen on G542X-CFTR. Figure 5A shows the western blot experiments of G542X-CFTR treated with type 1 corrector 605 (0.5 μM), type 2 corrector 990 (3 μM), and type 3 corrector VX445 (3 μM) with and without read-through reagent G418 (100 μM). The read-through G542X-CFTR protein or band C was barely visible when treated with DMSO, G418, or any one of the CFTR correctors marked (Figure 5A); however, the combination of correctors and G418 yielded a clear band C that is 6.6% ± 0.9%, 3.0% ± 0.8%, and 3.4% ± 0.5% of that of WT-CFTR for C1-605 + G418, C2-990 + G418 and C3-VX445 + 418, respectively (N = 3, Figure 5B).

As reported (Bridges et al., 2010; Lin et al., 2010), there is a synergistic effect between different types of the correctors for the protein expression of ΔF508-CFTR. As the read-through G542X-CFTR is responsive to all types of correctors (Figure 5B) in the presence of G418, we wonder if a similar synergism exists for G542X-CFTR. A variety of combinations of C1-605, C2-990, and C3-VX445 was tested with and without the presence of G418; however, no clear synergism was observed (Supplementary Figure S2). The read-through G542X-CFTR protein can only be detected with the presence of G418, and the protein expression when all three correctors were applied together was similar to that of C1-605 alone. These results outlined the fundamental difference between ΔF508 and PTC mutations such as G542X when designing the treatment strategy (see details in Discussion).

### Effects of Three Different Types of Correctors (C1-605, C2-990, and C3-VX445) on ΔF508-CFTR

**FIGURE 6:**
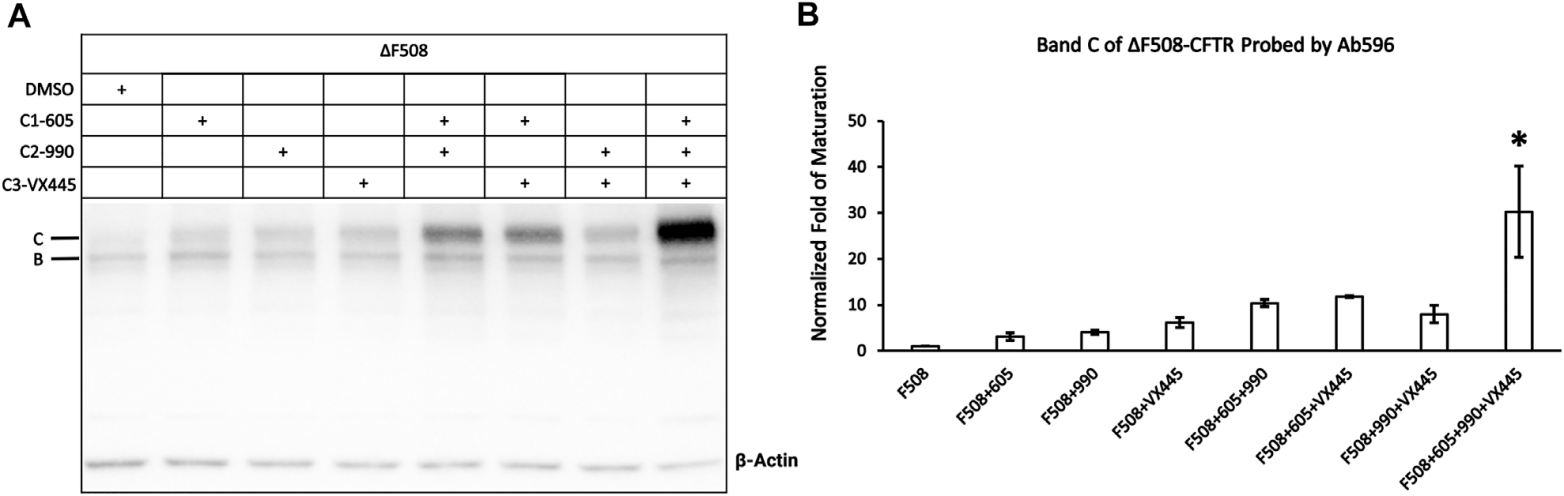
Western blot analysis of the expression of the ΔF508-CFTR. **(A)** Effects of type 1 (605), 2 (990), and 3 (VX-445) of CFTR correctors on ΔF508-CFTR expression. Gel was probed with antibody 596. **(B)** Quantification of the expression of the mature ΔF508-CFTR under different treatments compared to ΔF508 treated with DMSO. Data are represented as mean ± SEM. (N = 3, ANOVA followed by Tukey’s test). Triple combinations of C1-605, C2-990 and C3-VX445 have a significant increase compared to all other groups. *p < 0.05.

We next set out to determine whether these different correctors C1-605, C2-990, and C3-VX445 have synergistic effects on ΔF508-CFTR. Figure 6B shows that the expression of the mature ΔF508-CFTR was increased by <10-fold by C1-605, C2-990, and C3-VX445 alone, but more than 10-fold increase by the variety of two combinations of C1-605, C2-990, and C3-VX445. More significantly, the triple combination of C1-605, C2-990, and C3-VX445 increased the expression level of the mature ΔF508-CFTR 30-fold compared to that with DMSO, which is in line with the previous reports (Bridges et al., 2010; Lin et al., 2010) that combinations of different types of correctors exhibit synergistic effects on the biogenesis of ΔF508-CFTR.

### Whole-Cell Recordings of Cells Expressing G542X-CFTR

**FIGURE 7:**
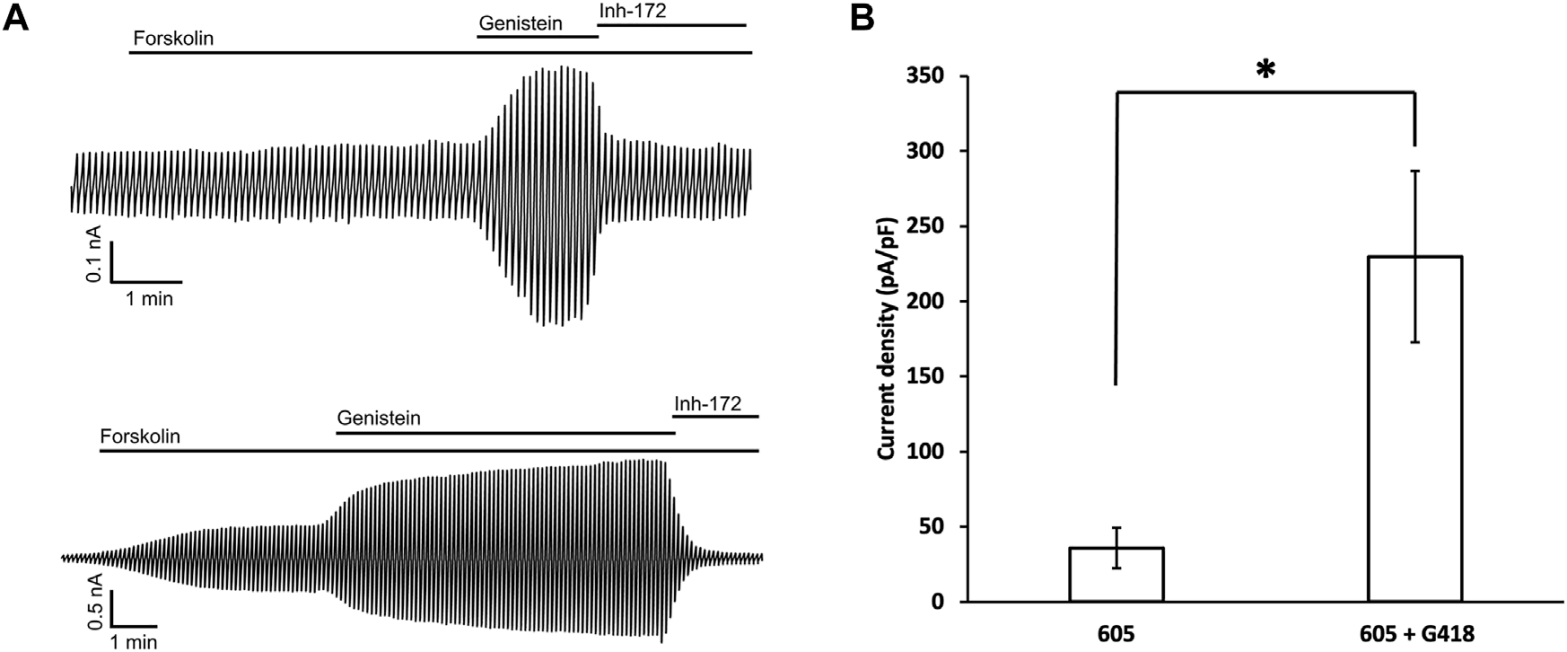
Whole-cell recordings of G542X-CFTR. **(A)** Whole-cell recordings of G542X-CFTR with incubation in 0.5 μM C1-605 (upper) and the combination of 0.5 μM C1-605 plus 100 μM G418 (lower). The currents were activated by 10 μM forskolin, potentiated by 30 μM genistein, and inhibited by 10 μM CFTRinh-172 (Inh-172). The membrane potential was held at 0 mV and a 200-ms voltage pulse over ± 100 mV was applied every 6 s. **(B)** Quantification of the current densities (at 100 mV) in forskolin plus genistein in the cells incubated with C1-605 (N = 7) and C1-605 + G418 (N = 16). The data are represented as mean ± SEM. *p < 0.05 by Student’s *t*-test.

To verify that the read-through proteins of G542X-CFTR can yield cAMP-dependent chloride currents, whole-cell recordings were conducted using the same protocol described in the previous study (Yeh and Hwang, 2020). 10 μM forskolin was first applied to cells treated with G418 and C1-605 overnight, once the amplitude of the forskolin-activated currents was increased to a stable level, 30 μM genistein was added as a CFTR potentiator to further increase the currents (Hwang et al., 1997), and then 10 μM CFTRinh-172 was used to ensure that the currents observed are indeed from CFTR (Kopeikin et al., 2010). C1-605 (Upper trace, Figure 7A) and the combination of C1-605 and G418 (Lower trace, Figure 7A) were chosen for the current whole-cell electrophysiological experiments (Figure 7), as the expression of Band C in G542X-CFTR treated with the combination of C1-605 and G418 is significantly increased compared to other treatments such as DMSO, C1-605, and G418 alone (Figures 1, 5). As shown in Figure 7, under the potentiation of 30 μM genistein, the current density of G542X incubated with C1-605 (Figure 7B) was similar to that of G542X incubated with either DMSO, VX-809, or G418 alone reported previously (Yeh and Hwang, 2020). However, when incubated with both C1-605 and G418, the current density under 30 μM genistein was about 5-fold for G542X compared to that of C1-605 (Figure 7B). The result is consistent with the western blot that the expression of G542X-CFTR band C was increased by the combination of the C1 corrector and the read-through reagent G418, corroborating the ideal that band C in Figure 1 is the functional read-through protein of G542X-CFTR, while the truncated protein of G542X-CFTR is not functional.

### Single-Channel Studies of G542X-CFTR

**FIGURE 8:**
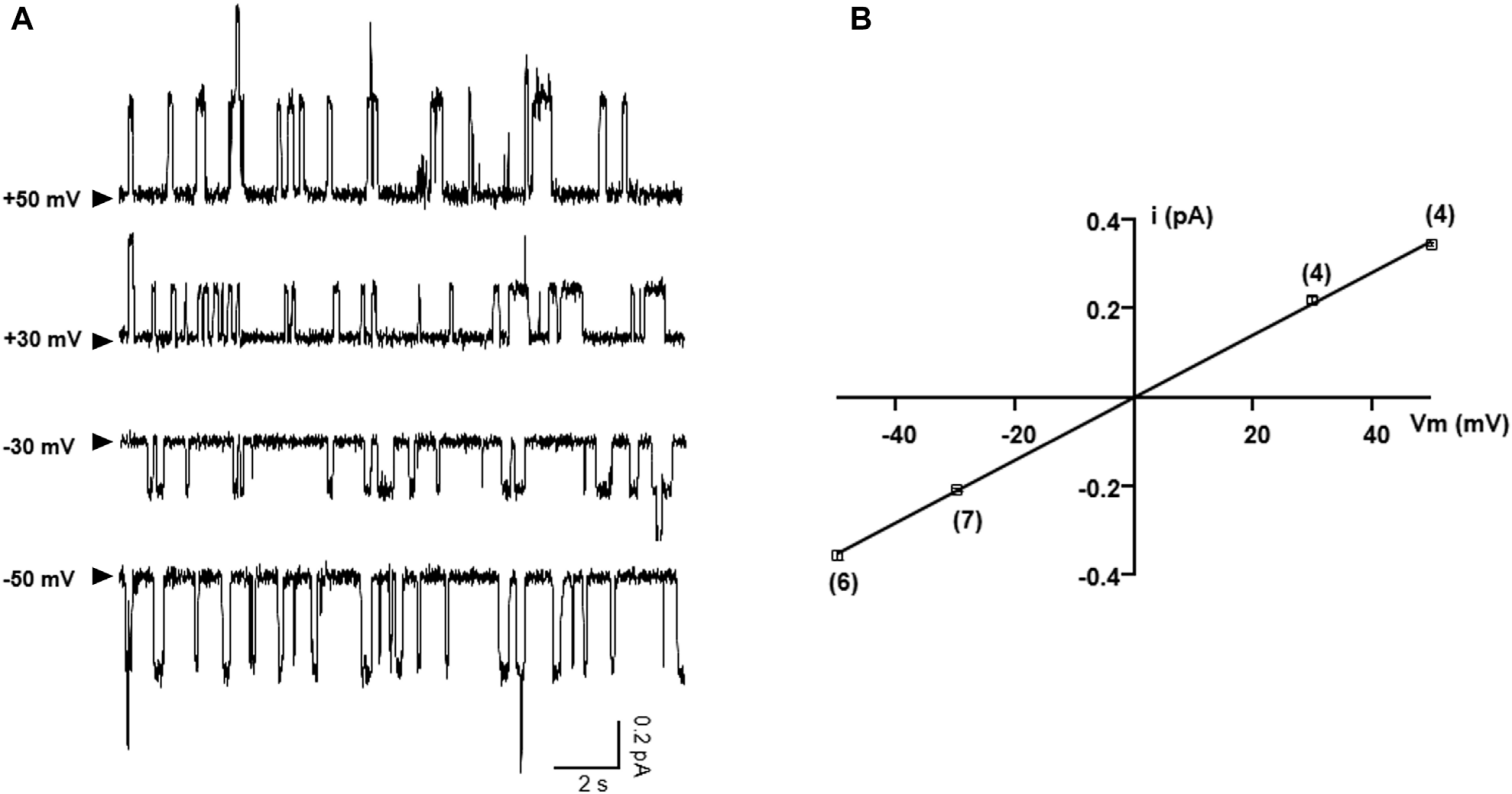
Single-channel *I-V* relationship of G542X-CFTR treated with DMSO. **(A)** Representative single-channel current traces of G542X-CFTR at different holding potentials. The arrowheads indicated that the baseline (closed state). **(B)** Single-channel *I-V* plot of G542X-CFTR, where the horizontal axis is the membrane potential (Vm), and the vertical axis is the single-channel amplitude (I). The squares are mean single-channel current amplitudes with the error bars representing SEM and the numbers of observations are indicated in the squares. The black line represents a linear fit of the single-channel *I-V* relationship of G542X-CFTR, yielding a single channel conductance of 6.75 pS, which is similar to that of WT-CFTR (Yeh et al., 2015).

As western bot and whole-cell recording experiments show that the functional read-through proteins of G542X-CFTR can be increased by combinations of correctors plus read-through agent G418, we further performed single-channel experiments to gain a deeper understanding of the read-through protein of G542X. Figure 8A shows representative single-channel recordings of G542X-CFTR at various membrane potentials. The resulting single-channel current-voltage (I-V) relationship of G542X-CFTR treated with DMSO reveals a single-channel conductance of 6.75 *pS* (Figure 8B), which is similar to that of WT (Yeh et al., 2015) or E60X-CFTR (Yeh and Hwang, 2020), supporting our prior study (Yeh and Hwang, 2020) that the read-through products in both G542X-CFTR and E60X-CFTR possess an intact chloride permeation pore similar to that of WT-CFTR channels.

**FIGURE 9:**
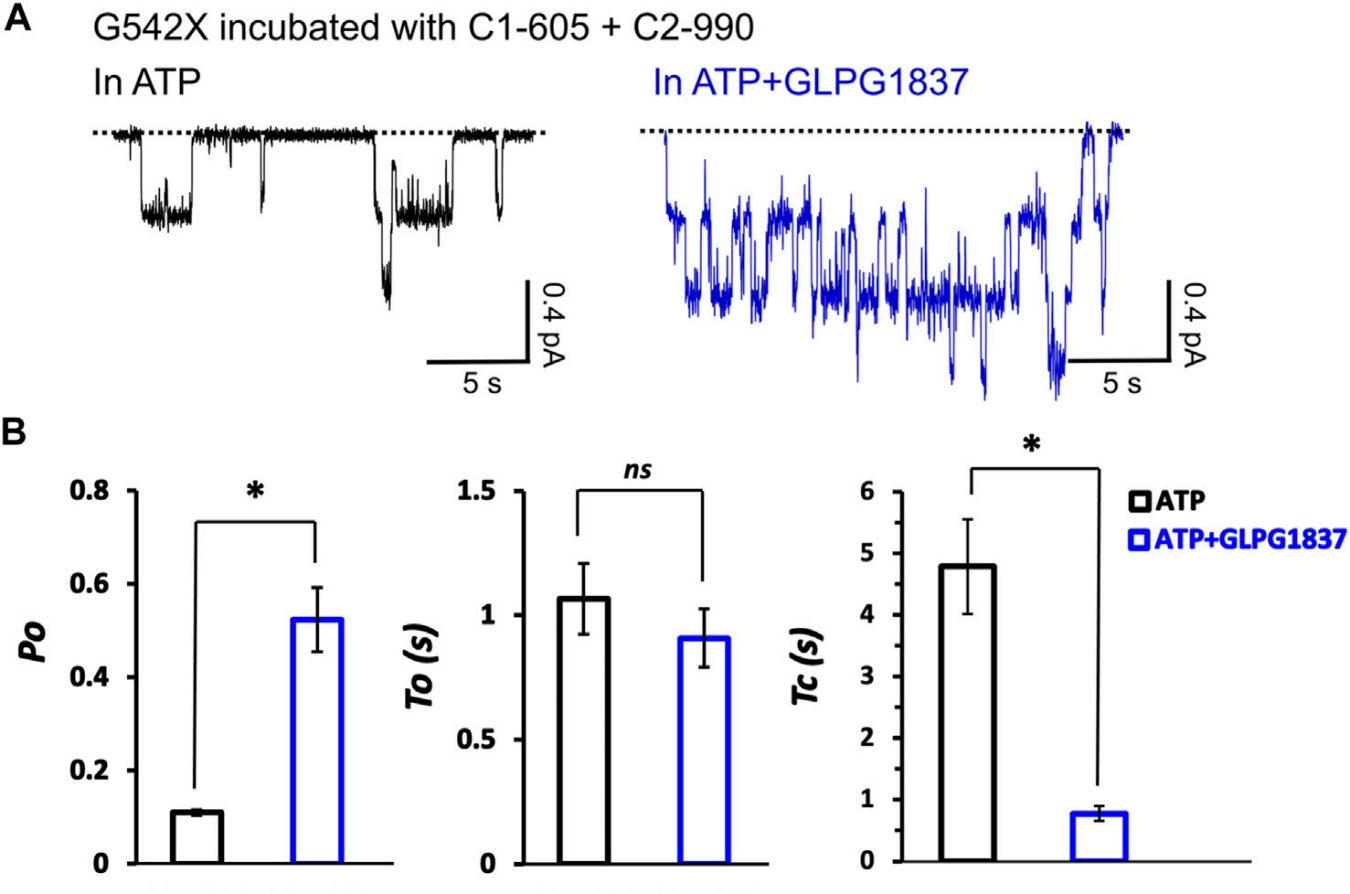
Single-channel behaviors and responses to channel modulators for G542X-CFTR read-through protein. **(A)** Single-channel recordings of G542X-CFTR treaded with and C1-605 (0.5 μM) + C2-990 (3 μM), respectively, in the presence of 2 mM ATP (black) and 2 mM ATP +3 µM GLPG 1837 (blue). **(B)** Statistics of open probability (*Po*), open time (*τ*_*o*_), and closed time (*τ*_*c*_). The data are represented as mean ± SEM. (N = 3, Student’s *t*-test). *p < 0.05. *ns* presents no significant increase.

We next examined the effects of incubation of C1-605 (0.5 μM) plus C2-990 (3 μM) on the kinetic properties of the read-through products of G542X-CFTR in ATP (2 mM) or ATP (2 mM) plus potentiator GLPG 1837 (3 μM) (Figure 9A). The *Po* of G542X-CFTR incubated with C-605 plus C2-990 in ATP is similar to that of the ones incubated with VX-809 reported previously (Yeh and Hwang, 2020), suggesting these correctors may not influence the gating of the channel. The *Po* increased by potentiator GLPG1837 by ∼ 6-fold, mainly due to shortening of the closed time (*τ*_c_) (Figure 9B). Unlike ΔF508-CFTR whose gating could be enhanced by some correctors (Okiyoneda et al., 2013), our single channel data suggested that the gating properties of G542X-CFTR are determined by the intrinsic nature of the read-through protein, which contains a missense mutation at position 542.

## Discussion

### Summary

Class I mutations such as the G542X-CFTR pose significant hurdles for developing CF treatment due to their multi-faceted deficits that could not be easily mitigated by the CFTR modulators used in clinics. However, the search for the most efficacious combination of CFTR correctors and potentiators is critical since reagents targeting the Class I pathogenesis such as read-through reagents or NMD inhibitors are still preliminary. In this study, we identified and characterized the C-terminus truncated protein and read-through protein of G542X-CFTR using different CFTR antibodies and mass-spectrometry, and examined their pharmacological responses to a range of reagents with different mechanisms either alone or in combination. We found that, unlike the most common disease-causing mutation ΔF508-CFTR, the combination of different types of CFTR correctors exerts a limited synergistic augmentation of the protein expression for the read-through G542X-CFTR. Also, consistent with our previous investigation (Yeh and Hwang, 2020), read-through reagents such as G418 are the prerequisite to producing enough full-length mature CFTR whose function can be further improved by CFTR potentiators.

### Characterization of the C-Terminus Truncated G542X-CFTR

In this study, we detected the C-terminus truncated G542X-CFTR using antibody MM13-4 recognizing the N-terminus (a.a. 25–36) of the protein (Figure 1A), and further confirmed its identity using the MS technique. Of all three types of CFTR correctors, only Type I corrector (C1-605) increased the expression of the C-terminus truncated protein (Figure 1), which is consistent with the recently-published cryo-EM study proposing that the binding site of type I correctors is located in the TMD1 (Fiedorczuk and Chen, 2022). Also, the novel corrector C1-605 likely shares a similar binding pocket in TMD1 with the FDA-approved type I corrector VX-809 and VX-661. As shown in Figure 4, the modification of amino acids involved in forming chemical bonds with VX-809 and VX-661 also impaired the effect of C1-605 (Fiedorczuk and Chen, 2022). Unlike C1 correctors, the binding sites of C2 and C3 are proposed to be NBD2 and NBD1, respectively (Okiyoneda et al., 2013), while the C-terminus truncated protein of G542X-CFTR only contains TMD1 and partially NBD1, it’s expected that C2 and C3 correctors do not influence the expression of this truncated protein. Despite the ability of C1-605 to increase the expression of the C-terminus truncated G542X-CFTR by about three-fold (Figure 3), this peptide containing the first 541 amino acids of CFTR is non-functional. Structurally, the truncated protein only possesses the TMD1 and a part of NBD1, which is unable to construct an ion permeation pathway. Indeed, although the expression of the C-terminus truncated G542X-CFTR was increased three-fold, pretreatment with C1-605 did not increase the whole-cell current compared to DMSO (Figure 7).

### Properties of the Read-Through G542-CFTR

We failed to detect the full-length read-through G542X-CFTR in our previous study probably due to insufficient sensitivity of the western blotting and a lack of reagents that increase the protein expression (Yeh and Hwang, 2020). In this study, we detected the read-through G542X-CFTR potentially due to several advancements in the experiments. First, we changed the transfer process from semi-dry to wet transferring (see Materials and Method), which may have a higher throughput (Mahmood and Yang, 2012). Second, instead of using VX-890 as the C1 corrector in the last study, we tested several novel correctors and optimized their concentration, which may lead to a higher efficacy for read-thorugh G542X-CFTR. Even with these improvements, the full-length read-through G542X-CFTR could only be detected with the combination of the correctors and read-through reagent G418, which is consistent with the notion that spontaneous or drug-assisted read-through is the rate-limiting step for the production of mature G542X-CFTR proteins (Figure 5).

The combination of different types of correctors demonstrated clear synergistic benefits for the ΔF508-CFTR (Bridges et al., 2010; Lin et al., 2010; Middleton et al., 2019). The combination of the three types of correctors in our study also yielded an up to 30-fold increase in the expression of the band C of ΔF508-CFTR (Figure 6). However, similar synergism was absent for the read-through proteins of G542X-CFTR. As shown in Supplementary Figure S2, the combination of all three types of correctors neither resulted in a detectable read-through protein signal in the absence of the read-through reagent nor increased the protein expression with the co-incubation of the read-through reagent compared to that of C1-605 alone. One of the interpretations of these results is that although all three types of the correctors bind to the read-through G542X-CFTR, the total amount of the protein produced by the G418-facilitated read-through is still too low to demonstrate a significant difference in expression with the combination of correctors.

Another possibility between the differential response for G542X-CFTR and ΔF508-CFTR is that, unlike ΔF508-CFTR which possesses a multifaceted folding and maturation defect, the only problem influencing the protein translation is the read-through process over the premature stop codon located in the TMD1. The introduction of this PTC in theory could influence the protein folding through the insertion of a missense mutation (or a set of missense mutations) and resulting in erroneous protein folding and structure. However, as our group has shown recently (Yeh and Hwang, 2020), mutating G542 to G542C and G542R had a minimum effect on full-length CFTR expression, suggesting that the read-through proteins out of G542X-CFTR may not exhibit defects of folding and maturation. Nonetheless, the read-through process itself may hinder the co-translational folding of the CFTR protein due to a decrease in translation speed by mechanisms such as ribosome stalling (Karamyshev and Karamysheva, 2018). As proposed in the recent structural study (Fiedorczuk and Chen, 2022), the binding of the Class I corrector stabilizes the TMD1 and makes it less likely to be degraded by the quality control system, and facilitating co-translation folding for the protein. We speculated that for the read-through G542X-CFTR, stabilizing the TMD1 by C1 is sufficient to promote the biogenesis process, whereas a further combination of C2 and C3 has a little additive effect.

From a functional aspect, the macroscopic current carried by the CFTR in a cell equals the product of the number of channels (N), the open probability, (*Po*) and single-channel conductance (i). Since the C-terminus truncated G542X-CFTR is non-functional, the maximally-potentiated macroscopic current (incubated with C1-605 plus G418 and under forskolin plus genistein) that is around 20% of that of WT-CFTR (Figure 7) is likely produced by the read-through G542X-CFTR. The single-channel recordings in Figures 8, 9 showed that the read-through G542X-CFTR has a single-channel conductance similar to that of WT, and a *Po* about 25% of that of WT. From these measurements, we can back-calculate the number of functional channels of G542X-CFTR pretreated with G418 and C1-605. The resulting baseline protein expression of 5%–10% of that of WT is consistent with the data in western blot experiments (Figures 1, 5). As there is limited room for further improvement of the *Po* or single-channel conductance, the bottleneck for mitigating the functional defect of G542X-CFTR is the low number of functional proteins.

### Therapeutic Implication for G542X-CFTR

The approval of triple combination therapy consisting of two types of correctors and a potentiator has launched a new era for CF therapy. The significant clinical efficacy of the combination therapy for patients with heterozygous or homozygous ΔF508 mutation (Middleton et al., 2019; Veit et al., 2020; Barry et al., 2021; Sutharsan et al., 2022) is now benefiting ∼90% of the CF patients. However, the rest 10%, mostly carrying PTC mutations, remain to be reached. We previously reported that not all PTC mutations are created equal; some face harsher therapeutic challenges. An important factor delineating the ideal treatment strategy for the Class I mutations is the position of the PTC. Depending on the functionality of C-terminus truncated proteins, PTC mutations should be further classified into two groups. When the PTC is located at a position after the complete construction of the ion conduction pathway (e.g., W1282X), the C-terminus truncated proteins can assume a residual function that can be further improved by the present CFTR modulators including the triple combination without the help of read-through reagents. The reports have indeed shown that the combinations of C1-VX809 + potentiator VX770 or C1a + C2a + potentiator GLPG 1837 (Haggie et al., 2017; Mutyam et al., 2021) are efficacious for W1282X. But for PTCs like G542X whose C-terminus truncated proteins are nonfunctional, the traditional combination of CFTR correctors and potentiators will not work unless enough full-length proteins are produced by an efficacious read-through. Furthermore, since missense mutations invariably occur during the read-through process, the read-through proteins from a PTC mutation likely exhibit additional deficits. A comprehensive investigation of the properties of the read-through proteins will thus offer insights into directing the treatment strategies. In conclusion, our study demonstrated the pharmacological responses of the C-terminus truncated and read-trough G542X-CFTR proteins to a range of novel CFTR modulators and read-through reagent G418 in different combinations, and highlighted the need for individual characterization of the PTC mutation to help guide the development of the most effective treatment strategies.

## Data Availability

The original contributions presented in the study are included in the article/Supplementary Material; further inquiries can be directed to the corresponding author.

## Supplementary Material

Supplementary Figure S1 Mass spectroscopic characterization of proteins translated from G542X-CFTR. **(A)** Identified peptides, shown with blue underline using SDS as the elution buffer after immunoprecipitation, cover 53% of the WT-CFTR sequence and 44% of the C-terminus truncated G542X-CFTR sequence. **(B)** Identified peptides, shown with blue underline using the glycine buffer (pH = 2.5) as the elution buffer after immunoprecipitation, cover 50% of the WT-CFTR sequence, and 44% of the C-terminus truncated G542X-CFTR sequence.

Supplementary Figure S2 Western blot analysis of the expression of the read-through proteins of G542X-CFTR using antibody 596 which recognizes CFTR’s NBD2. **(A)** Effects of type 1 (605), 2 (990), and 3 (VX-445) CFTR correctors with or without the read-through reagent G418 on G542X-CFTR expression. **(B)** Quantification of the expression of the read-through proteins of G542X-CFTR treated with type 1 (605), 2 (990), 3 (VX-445), and the read-through agent G418 compared to WT-CFTR. The data are presented as mean ± SEM. The asterisks indicated that the expression of the read-through proteins is significantly higher when treated with correctors and the read-through agent G418 compared with DMSO (N = 3, ANOVA followed by Dunnett's test). **p* < 0.05; ***p* < 0.01. **(C)** Quantification of the expression of the read-through proteins of G542X-CFTR treated with all correctors investigated in this study and the read-through agent G418 compared to WT-CFTR. Multiple comparisons were not applied in **(C)** since those multiple experiments were not carried out at the same time.
