# Supplementary figures and images for: Pharmacological Responses of the G542X-CFTR to CFTR Modulators
Source: Front Mol Biosci. 2022 Jun 24;9:921680. doi: 10.3389/fmolb.2022.921680 (PMC9263564; doi:10.3389/fmolb.2022.921680)

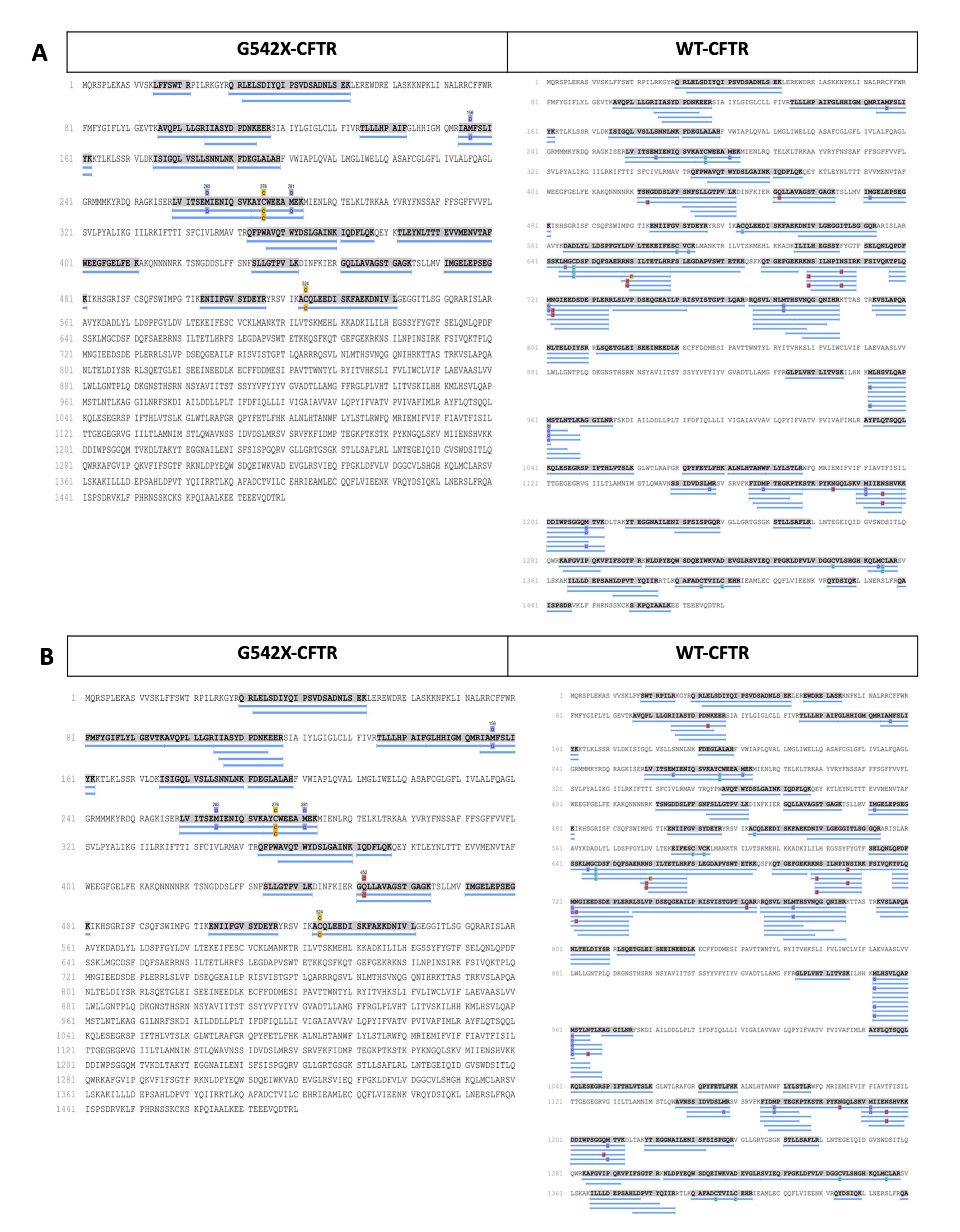

Supplement: Supplementary file 1 [file Image1.tiff]

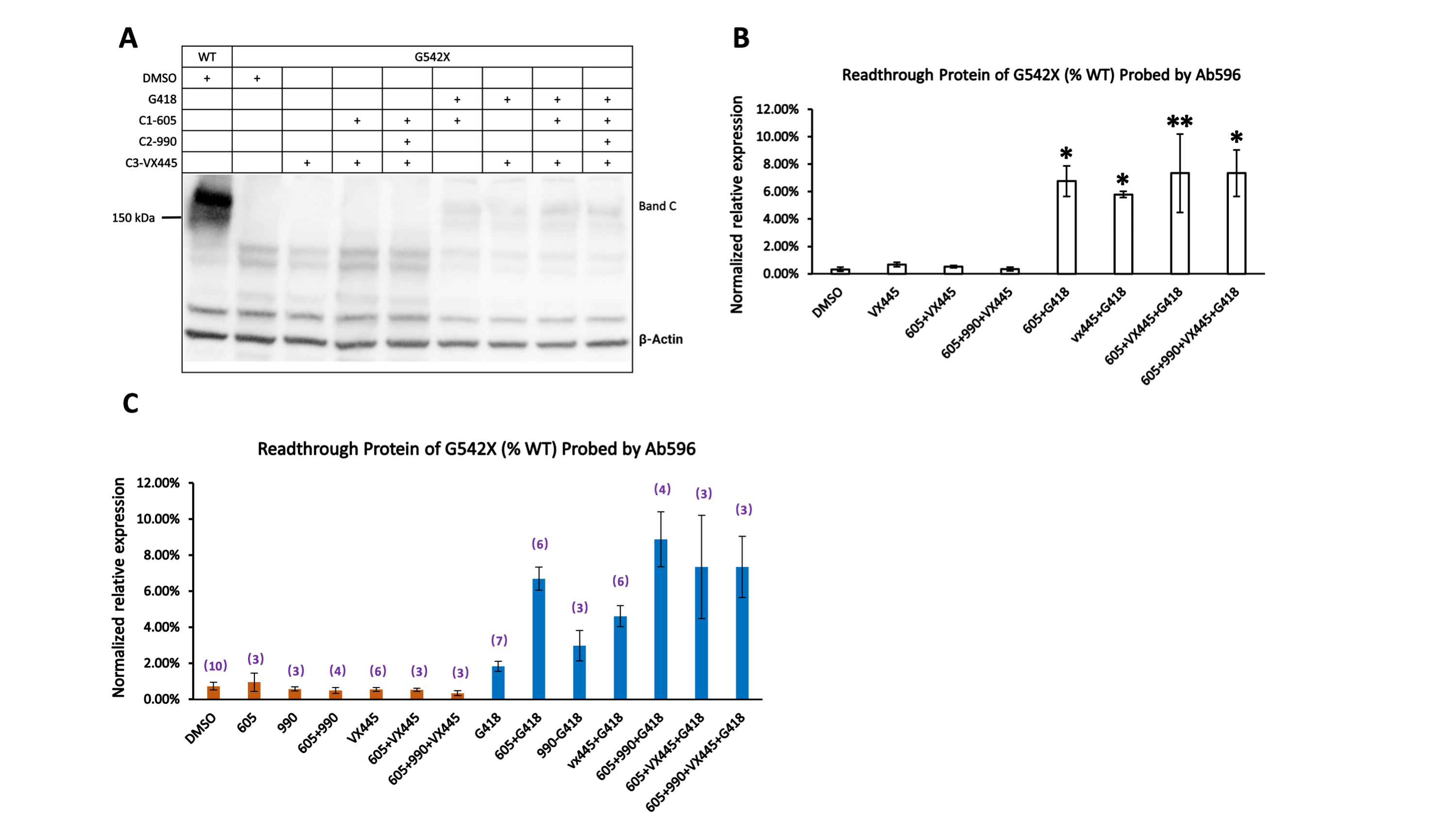

Supplement: Supplementary file 2 [file Image2.tiff]
